# The mediating role of psychological capital in the association between life satisfaction and depressive and anxiety symptoms among Chinese medical students during the COVID-19 pandemic: a cross-sectional study

**DOI:** 10.1186/s12888-023-04894-7

**Published:** 2023-06-05

**Authors:** Simeng Wang, Honghe Li, Xin Chen, Nan Yan, Deliang Wen

**Affiliations:** 1 Institute for International Health Professions Education and Research, China Medical University, No. 77 Puhe Road, Shenyang North New Area, Shenyang, 110122 Liaoning PR China; 2 Department of Epidemiology, School of Public Health, Dalian Medical University, No. 9 West Section of Lvshun South Road, Lvshunkou District, Dalian, 116044 Liaoning PR China; 3 School of Medical Applied Technology, Shenyang Medical College, No.146 Huanghe Street, Yuhong District, Shenyang, 110034 Liaoning PR China

**Keywords:** Depressive symptoms, Anxiety symptoms, Life satisfaction, Psychological capital, COVID-19, Medical students

## Abstract

**Background:**

Although life satisfaction is a predictor of depressive and anxiety symptoms, the mechanisms underlying this association are poorly understood. This study examined how psychological capital (PsyCap), a positive psychological state, mediated the association between life satisfaction and depressive and anxiety symptoms among Chinese medical students during the COVID-19 pandemic.

**Methods:**

A cross-sectional survey was conducted at three medical universities in China. A self-administered questionnaire was distributed to 583 students. Depressive symptoms, anxiety symptoms, life satisfaction, and PsyCap were measured anonymously. A hierarchical linear regression analysis was performed to explore the effects of life satisfaction on depressive and anxiety symptoms. Asymptotic and resampling strategies were used to examine how PsyCap mediates the association between life satisfaction and depressive and anxiety symptoms.

**Results:**

Life satisfaction was positively associated with PsyCap and its four components. There were significant negative associations between life satisfaction, psychological capital, resilience, optimism, and depressive and anxiety symptoms among medical students. Self-efficacy was negatively associated with depressive and anxiety symptoms. Psychological capital (a×b = -0.3201, BCa 95% CI: -0.3899, -0.2446; a×b = -0.2749, BCa 95% CI: -0.3817, -0.1996), resilience (a×b = -0.2103, BCa 95% CI: -0.2727, -0.1580; a×b = -0.1871, BCa 95% CI: -0.2520, -0.1414), optimism (a×b = -0.2100, BCa 95% CI: -0.3388, -0.1150; a×b = -0.1998, BCa 95% CI: -0.3307, -0.0980), and self-efficacy (a×b = -0.0916, BCa 95% CI: 0.0048, 0.11629; a×b = 0.1352, BCa 95% CI: 0.0336, 0.2117) significantly mediated the association between life satisfaction and depressive and anxiety symptoms.

**Limitations:**

This was a cross-sectional study, and causal relationships between the variables could not be ascertained. Self-reported questionnaire instruments were used for data collection, which may have recall bias.

**Conclusions:**

Life satisfaction and PsyCap can be used as positive resources to reduce depressive and anxiety symptoms among third-year Chinese medical students during the COVID-19 pandemic. Psychological capital and its components (self-efficacy, resilience, and optimism) partially mediated the relationship between life satisfaction and depressive symptoms, and completely mediated the relationship between life satisfaction and anxiety symptoms. Therefore, improving life satisfaction and investing in psychological capital (especially self-efficacy, resilience, and optimism) should be included in the prevention and treatment of depressive and anxiety symptoms among third-year Chinese medical students. Additional attention is needed to pay for self-efficacy in such disadvantageous contexts.

**Supplementary Information:**

The online version contains supplementary material available at 10.1186/s12888-023-04894-7.

## Background

A pandemic not only affects physical health, but the large-scale spread of infection can also affect people’s mental health due to pandemic-associated anxiety and fear [1]. Moreover, in the context of a pandemic, public health measures such as social distancing and lockdowns can have negative psychological effects. Depression is a major public health problem characterised by sadness, decreased energy, and loss of interest in activities. Depression severity, symptoms, and duration differ from those of normal mood changes [2]. Anxiety is a vague feeling of apprehension, worry, restlessness, or fear, the sources of which are often non-specific or unknown to the individual [3]. It is well known that medical education is often more intensive and takes longer to complete than many other courses. Thus, it is not surprising that medical students have a higher risk of depressive and anxiety symptoms compared to the general student population [4, 5].

The prevalence of depressive and anxiety symptoms varies across universities and countries, with reported anxiety symptoms prevalence ranging from 7.7 to 65.5% and depressive symptoms prevalence ranging from 6 to 66% [4–6]. Medical students exhibit higher levels of anxiety symptoms, depressive symptoms, and stress than the general population [7]. Depressive symptoms affect one in three medical students worldwide, according to a recent meta-analysis [4]. Furthermore, approximately 30% of medical students in Europe suffer from either depressive or anxiety symptoms [8, 9]. Symptoms of anxiety and depression can adversely affect medical students, leading to dropping out of school, poor academic performance, drug and alcohol abuse, and suicidal ideation and attempts [10]. Therefore, it is important to pay attention to anxiety and depressive symptoms among medical students, especially during the COVID-19 pandemic.

Life satisfaction refers to a person’s general assessment of their own mental health and quality of life; it includes emotional and cognitive evaluations of their personal lives [11]. Life satisfaction is frequently used to define quality of life and happiness [12]. Furthermore, it is considered a key metric of quality of life (QOL) and a significant aspect of positive psychology [13]. Individuals with high life satisfaction generally exhibit a more positive mental state, associated with less stress and fewer anxiety symptoms [14]. Moreover, higher levels of life satisfaction are linked to better health, job performance, and social development [15]. Recently, life satisfaction has received considerable research attention, both locally and internationally, especially among students [12]. One’s student life is a time of positive professional and personal self-determination. One criterion for success in self-determination is a high level of life satisfaction [16]. According to previous research [17], in addition to having a direct effect on anxiety and depressive symptoms, life satisfaction has an indirect effect through the activation of specific psychological responses. Thus, the indirect and direct effects of life satisfaction on these symptoms should be investigated further to develop effective prevention strategies for depressive and anxiety symptoms.

Positive psychological capital (PsyCap) is a high-level core texture in line with positive organizational behaviour, proposed by the research and application of positive-oriented human resource advantages and psychological capabilities [18]. PsyCap comprises four state-based psychological resource competencies—resilience, self-efficacy, optimism, and hope—all of which can be measured effectively [19]. Resilience is a positive mental ability that enables recovery from failure and adversity, leading to success. Optimism is a positive manner of explaining successful self-attribution [20]. Self-efficacy is a positive belief in one’s ability to successfully complete challenging tasks. Hope is a positive state that directs individuals’ will towards desired goals and places them on the road to success. In a previous study, PsyCap worked as a positive resource against depressive symptoms, mediating the relationship between occupational stress and depressive symptoms among Chinese physicians [21]. In another study, the mediating role of PsyCap was supported for job attitudes [22]. A study in the Philippines found out that PsyCap was positively correlated with students’ life satisfaction [23]. Wang et al. [24] pointed out that improving college students’ PsyCap could help reduce depressive and anxiety symptoms in this population. However, whether PsyCap mediates the association between life satisfaction and symptoms of anxiety and depression among medical students remains to be established. Understanding the influence of PsyCap on this relationship is important for effective prevention and treatment of anxiety and depressive symptoms among Chinese medical students.

Considering the above, this study investigated anxiety and depressive symptoms among third-year undergraduates majoring in clinical medicine at three medical schools, during the COVID-19 pandemic. The aims of this study were threefold: first, to examine the association between life satisfaction and PsyCap; second, to determine the relationship between PsyCap and symptoms of anxiety and depression; and third, to investigate whether PsyCap mediates the relationship between life satisfaction and symptoms of anxiety and depression among Chinese medical students.

## Methods

### Research design and sample

A two-month cross-sectional survey was initiated in November 2021, recruiting third-year medical undergraduates from China Medical University, Dalian Medical University, and Shenyang Medical College. Using a power of 80%, a confidence level of 95%, and a margin of error of 5%, the estimated sample size was set to 383 using the following formula: X = Z_α/2_^2^ × *p* × (1 − p)/MOE^2^ [25, 26]. To account for incomplete questionnaires (up to 20%), the minimum number of participants was increased to 479. Based on the sample size obtained in the early stages and the respective situations of the three universities, several classes were randomly sampled from third-year undergraduate students majoring in clinical medicine at each university in the same proportion; 616 medical students were recruited and 603 questionnaires were collected. Twenty questionnaires contained obvious errors and incorrect or incomplete answers to the polygraph questions. Effective responses were received from 583 students, with an effective response rate of 94.64%. This study used an online survey that was anonymously filled and did not involve private and sensitive topics. The survey was conducted through an online survey platform called ‘Questionnaire Star’, and the point of contact sent a direct link to the students who volunteered to fill out the questionnaire. The questionnaire system provided rewards to encourage students to participate and ensure the quality of responses. This study complied with the requirements of the ethics committee.

### Measuring instruments

#### Center for epidemiologic studies depression scale (CES-D)

Depressive symptoms were assessed by the 20-item Chinese version of the Center for Epidemiologic Studies Depression Scale (CES-D) [27, 28]. Each item consisted of four options describing the frequency of each feeling in the past week, ranging from 0 (rarely or none of the time [less than one day]) to 3 (all of the time [five to seven days]). The CES-D has been validated in a variety of Chinese samples. Among college students, it had a Cronbach’s alpha of 0.87, which is acceptable [29]. Cronbach’s alpha for the CES-D for medical students in this study was 0.95.

#### Self-rating anxiety scale (SAS)

The Chinese version of the Self-Rating Anxiety Scale (SAS) was used to assess anxiety symptoms in college students during the past week [30]. The SAS consists of 20 items rated on a 4-point Likert scale ranging from 1 = none or very little (< 1 day) to 4 = most or all (5–7 days). Of these items, 5, 9, 13, 17, and 19 are reverse-graded. The SAS has good internal consistency and reliability in the Chinese population [30, 31], and the Cronbach’s alpha coefficient in this study was 0.92.

#### Satisfaction with life scale (SWLS)

Life satisfaction was examined using the Satisfaction with Life Scale (SWLS) developed by Diener et al. [32]; it consists of five items scored on a 7-point Likert scale (1 = strongly disagree, 2 = disagree, 3 = slightly disagree, 4 = neither agree nor disagree, 5 = slightly agree, 6 = agree, and 7 = strongly agree). The total score is calculated using the summed aggregate items. This scale is reliable for cross-cultural purposes. Diener et al. [33] reported the reliability and validity of the scale with a Cronbach’s alpha of 0.80. In this study, the SWLS had a Cronbach’s alpha of 0.905.

#### Positive psychological capital questionnaire (PPQ)

A positive PsyCap questionnaire was used to assess the medical students’ PsyCap. This questionnaire was developed by Zhang et al. [34] based on the PsyCap Questionnaire (PCQ) compiled by Luthans et al. [35]. The questionnaire contains 26 items and 4 dimensions: self-efficacy, resilience, hope, and optimism. A Likert-type scale is used to evaluate each item. The participants choose from complete disagreement to complete agreement based on the actual situation. Each item is scored from 1 to 7. The overall Cronbach’s alpha coefficient for the questionnaire was 0.946, and the Cronbach’s alpha coefficients for each dimension were 0.928, 0.733, 0.922 and 0.845, respectively.

### Demographic characteristics

Demographic information regarding place of residence, sex, only-child, parental education status, and exercise were obtained. Place of residence was categorised as urban or rural. The parental educational level was categorised as junior high or lower, senior high school, or junior college or higher.

### Statistical analysis

**Fig. 1:**
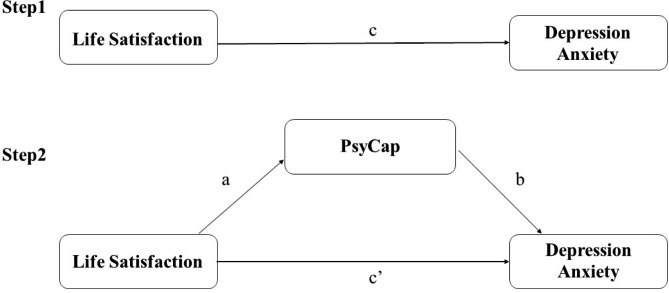
Theoretical model of the mediating role of PsyCap on the association between life satisfaction and depressive and anxiety symptoms. c: associations of life satisfaction with depressive and anxiety symptoms; a: associations of life satisfaction with PsyCap; b: association between PsyCap and depressive and anxiety symptoms after controlling for the predictor variables; c’: associations of life satisfaction with depressive and anxiety symptoms after adding PsyCap as a mediator

The descriptive statistics of the study variables were appropriately represented as mean, standard deviation (SD), number (N), or percentage (%). Independent samples t-test and one-way analysis of variance (ANOVA) were used to examine the distribution of demographic factors for depressive and anxiety symptoms. When the one-way ANOVA was significant, multiple comparisons were performed. The correlations between continuous variables were tested using Pearson’s correlation coefficients. Hierarchical linear regression analysis was used to explore the links between life satisfaction and anxiety and depressive symptoms after adjustment (*p* < 0.05). Sex was added in Block 1, and life satisfaction in Block 2. Two models (Models 1 and 2) were used in Block 3; PsyCap was added to Model 1, and resilience, self-efficacy, optimism, and hope were added to Model 2. The mediating effects of PsyCap and its four components on life satisfaction and symptoms of depression and anxiety were investigated using asymptotic and resampling strategies (a×b product). In these equations, life satisfaction was the independent variable, CES-D and SAS scores were dependent variables, PsyCap and its components were intermediate variables (Fig. 1), and sex was a covariate. The bootstrap estimate was based on 1000 bootstrap samples. The bias-corrected and accelerated 95% confidence intervals (BCa 95% CI) for each a×b product were then investigated, excluding a BCa 95% CI of 0, indicating a significant mediating effect. All study variables were centrally processed prior to analysis to account for differences in scale scores. Data including R^2^ changes, adjusted R^2^, R^2^, standardisation regression coefficients (*β*), F, and *p* value were provided for each step in the regression model. Multicollinearity was tested using variance inflation factors (< 5) and tolerance (> 0.10). Statistical analysis was performed using the Statistical Package for the Social Sciences (SPSS, version 20.0), and a two-tailed probability value of < 0.05 was considered statistically significant.

## Results

### Participant characteristics

Of the 616 questionnaires sent, 583 (94.64%) were considered valid and suitable for analysis. Demographic characteristics are shown in Table 1. Female participants represented 55.4% of the sample, and 59.2% were from urban areas. The educational status of most of the students’ fathers was junior college or higher, and that of their mothers was junior high or lower. Most of the students (91.9%) did not exercise. In our research, the prevalence of anxiety symptoms among medical students in China was 13.2%, of which mild, moderate and severe anxiety symptoms were 10.8%, 2.2% and 0.2% respectively, and the prevalence of depressive symptoms was 72%. Male students were more likely to experience anxiety and depressive symptoms than female students (*p* < 0.05).

**Table 1:** Demographic variables and differences in Depressive and Anxiety symptoms (N = 583)

Variables	N	%	Depressive symptoms (SD)	t/F^a^	*p*	Anxiety symptoms (SD)	t/F^a^	*p*
**Sex**								
Male	260	44.6	21.86 (11.22)	2.347	0.019^*^	40.34 (10.37)	2.788	0.005^**^
Female	323	55.4	19.72 (10.69)			38.02 (9.72)		
**Place of residence**								
Urban	345	59.2	20.15 (11.68)	-1.389	0.165	38.44 (10.54)	-1.771	0.077
Rural	238	40.8	21.43 (9.84)			39.94 (9.31)		
**Only-child**								
Yes	325	55.7	20.78 (11.32)	0.249	0.803	39.16 (10.41)	0.287	0.774
No	258	44.3	20.55 (10.53)			38.92 (9.65)		
**Father’s education status**								
Junior high or lower	226	38.8	21.54 (9.85)	1.159	0.315	39.97 (9.59)	1.544	0.214
Senior high school	108	18.5	20.23 (10.61)			38.52 (9.43)		
Junior college or higher	249	42.7	20.08 (12.04)			38.45 (10.74)		
**Mother’s education status**								
Junior high or lower	246	42.2	21.17 (9.72)	0.623	0.537	39.46 (9.06)	1.09	0.337
Senior high school	114	19.6	20.85 (10.72)			39.69 (10.10)		
Junior college or higher	223	38.3	20.05 (12.34)			38.28 (11.07)		
**Exercise**								
No	536	91.9	20.78 (11.01)	0.816	0.415	39.12 (10.08)	0.551	0.582
Yes	47	8.1	19.43 (10.52)			38.28 (10.12)		

^a^ Independent sample t-test and one-way ANOVA were used

* *p* < 0.05; ** *p* < 0.01

### Correlations among continuous variables

As described in Table 2, anxiety and depressive symptoms were negatively correlated with life satisfaction (r = -0.439 to -0.338; *p* < 0.001), PsyCap (r = -0.549 to -0.445; *p* < 0.001), self-efficacy (r = -0.345 to -0.241; *p* < 0.001), resilience (r = -0.611 to -0.514; *p* < 0.001), hope (r = -0.432 to -0.361; *p* < 0.001), and optimism (r = -0.545 to -0.458; *p* < 0.001). Conversely, life satisfaction was positively correlated with PsyCap (r = 0.670; *p* < 0.001), self-efficacy (r = 0.553; *p* < 0.001), resilience (r = 0.445; *p* < 0.001), hope (r = 0.647; *p* < 0.001), and optimism (r = 0.659; *p* < 0.001).

**Table 2:** Descriptive statistics and zero-order correlations (Pearson’s r) among study variables

Variables	Mean (SD)	95% CI		Median	1	2	3	4	5	6	7	8
		Lower	Upper									
1. Depressive symptoms	20.68 (10.97)	19.79	21.57	22	1	0.854^***^	-0.439^***^	-0.549^***^	-0.345^***^	-0.611^***^	-0.432^***^	-0.545^***^
2. Anxiety symptoms	39.05 (10.08)	38.23	39.87	40		1	-0.338^***^	-0.445^***^	-0.241^***^	-0.514^***^	-0.361^***^	-0.458^***^
3. Life satisfaction	23.77 (6.16)	23.26	24.27	23			1	0.670^***^	0.553^***^	0.445^***^	0.647^***^	0.659^***^
4. PsyCap	122.62 (23.74)	120.69	124.55	121				1	0.882^***^	0.737^***^	0.898^***^	0.910^***^
5. Self efficacy	32.86 (8.16)	32.19	33.52	33					1	0.521^***^	0.741^***^	0.701^***^
6. Resilience	30.67 (6.20)	30.16	31.17	29						1	0.478^***^	0.589^***^
7. Hope	29.88 (6.89)	29.32	30.44	30							1	0.855^***^
8. Optimism	29.21 (6.34)	28.70	29.73	29								1

Abbreviations: PsyCap: Psychological capital; SD: Standard deviation; CI: Confidence interval

*** *p* < 0.001

### Associations of life satisfaction and PsyCap with depressive and anxiety symptoms

Table 3 presents the hierarchical regression analysis of depressive and anxiety symptoms after adjusting for covariates. Life satisfaction was negatively correlated with depressive (*β* = -0.438, *p* < 0.001) and anxiety symptoms (*β* = -0.338, *p* < 0.001), accounting for 19.3% and 11.4% of the variance in Block 2, respectively. In Block 3, in Model 1, life satisfaction (*β* = -0.119, *p* < 0.05) and PsyCap (*β* = -0.476, *p* < 0.001) were significantly negatively correlated to depressive symptoms, accounting for 12.4% of the difference in addition to life satisfaction. PsyCap was negatively correlated with anxiety symptoms (*β* = -0.410, *p* < 0.001), accounting for 9.2% of the difference in addition to life satisfaction. Furthermore, tolerance (range 0.547–0.994) and variance inflation (range 1.006–1.827) did not reveal significant multicollinearity issues. In Block 3 Model 2, life satisfaction (*β* = -0.124, *p* < 0.01), optimism (*β* = -0.322, *p* < 0.001), and resilience (*β* = -0.473, *p* < 0.001) were significantly negatively associated with depressive symptoms, while self-efficacy was positively correlated with depressive symptoms (*β* = 0.160, *p* < 0.001), accounting for 25.9% of the difference in addition to life satisfaction. Resilience (*β* = -0.425, *p* < 0.001) and optimism (*β* = -0.303, *p* < 0.001) were significantly negatively associated with anxiety symptoms, while self-efficacy was positively correlated with anxiety symptoms (*β* = 0.241, *p* < 0.001), accounting for 21.9% of the difference in addition to life satisfaction. Furthermore, compared with Block 2, life satisfaction in Block 3 had a smaller effect on anxiety and depressive symptoms, manifested by a smaller *β* coefficient. In addition, tolerance (range 0.214–0.964) and variance inflation (range 1.038–4.678) did not show significant multicollinearity issues.

**Table 3:** Results from the hierarchical multiple regression analyses

	Variables	Block 1 (β)				Block 2 (β)			Block 3 (β)	
									Model 1	Model 2
Depressive symptoms										
	Sex	-0.097***				-0.095*			-0.122***	-0.110***
	Life satisfaction					-0.438***			-0.119*	-0.124**
	PsyCap								-0.476***	
	Self-efficacy									0.160***
	Resilience									-0.473***
	Hope									0.031
	Optimism									-0.322***
	F	5.507*				73.251***			93.245***	82.010***
	Adjusted R^2^	0.008				0.199			0.322	0.455
	ΔR^2^	0.009*				0.192***			0.124***	0.259***
	Total R^2^	0.009				0.202			0.326	0.461
Anxiety symptoms										
	Sex	-0.115**				-0.114**			-0.137***	-0.115**
	Life satisfaction					-0.338***			-0.063	-0.054
	PsyCap								-0.410***	
	Self-efficacy									0.241***
	Resilience									-0.425***
	Hope									-0.043
	Optimism									-0.303***
	F	7.772**				42.280***			54.188***	50.787***
	Adjusted R^2^	0.012				0.124			0.215	0.339***
	ΔR^2^	0.013**				0.114***			0.092***	0.219***
	Total R^2^	0.013				0.127			0.219	0.346***

Abbreviations: Adj.R^2^: adjusted R^2^; β: standardized regression coefficient

* *p* < 0.05; ** *p* < 0.01; *** *p* < 0.001

### Mediating effects of PsyCap and its components

Path coefficients c (life satisfaction and symptoms of depression and anxiety), c’ (between life satisfaction, anxiety, and depressive symptoms after adding mediators), a (between life satisfaction and mediators), b (between mediators and anxiety and depressive symptoms), a×b products, and BCa 95% CI for these products are presented in Table 4. Life satisfaction was negatively correlated with depressive and anxiety symptoms among medical students (Path c), and significantly positively correlated with PsyCap and its components (self-efficacy, resilience, optimism, and hope; Path a). Consistent with the hierarchical multiple regression results, after controlling for sex and life satisfaction, PsyCap and its components (optimism and resilience) were negatively correlated with anxiety and depressive symptoms among medical students, whereas self-efficacy was positively correlated with anxiety and depressive symptoms (Path b). Thus, the results revealed significant mediating roles of PsyCap (a×b = -0.3201, BCa 95% CI: -0.3899, -0.2446), self-efficacy (a×b = 0.0916, BCa 95% CI: 0.0048, 0.1629), resilience (a×b = -0.2103, BCa 95% CI: -0.2727, -0.1580), and optimism (a×b = -0.2100, BCa 95% CI: -0.3388, -0.1150) in the link between life satisfaction and depressive symptoms among medical students. When PsyCap and its components were included as mediators in the model, the direct pathway between life satisfaction and depressive symptoms (Path c’) remained significant. Similarly, significant mediating roles of PsyCap (a×b = -0.2749, BCa 95% CI: -0.3817, -0.1996), self-efficacy (a×b = 0.1352, BCa 95% CI: 0.0336, 0.2117), optimism (a×b = -0.1998, BCa 95% CI: -0.3307, -0.0980), and resilience (a×b = -0.1871, BCa 95% CI: -0.2520, -0.1414) were found in the link between life satisfaction and anxiety symptoms among medical students. The direct pathway between life satisfaction and anxiety symptoms was not significant (Path c’). We also found mediating effects of PsyCap and its components in medical students with depressive (CES-D ≥ 16) and anxiety symptoms (SAS ≥ 50), as shown in Supplementary Appendix 1.

**Table 4:** Mediating roles of PsyCap and its components on the Life satisfaction-Depressive/Anxiety symptoms association

	Mediators	a			b		c		c’				a×b (BCa 95% CI)			R^2^
Depressive symptoms																
	PsyCap	0.6707^***^			-0.4762^***^			-0.4385^***^	-0.1191^**^					-0.3201^*^ (-0.3899, -0.2446)		0.3258
	Self-efficacy	0.5533^***^			0.1602^***^			-0.4385^***^	-0.1244^**^					0.0916^*^ (0.0048, 0.1629)		0.4607
	Resilience	0.4456^***^			-0.4725^***^									-0.2103^*^ (-0.2727, -0.1580)		
	Hope	0.6471^***^			0.0307									0.0181 (-0.0784, 0.1296)		
	Optimism	0.6592^***^			-0.3216^***^									-0.2100^*^ (-0.3388, -0.1150)		
Anxiety symptoms																
	PsyCap	0.6707^***^			-0.4099^***^			-0.3377^***^	-0.0628					-0.2749^*^ (-0.3476, -0.2110)		0.2192
	Self-efficacy	0.5533^***^			0.2413^***^			-0.3377^***^	-0.0544					0.1352^*^ (0.0336, 0.2117)		0.3460
	Resilience	0.4456^***^			-0.4248^***^									-0.1871^*^ (-0.2520, -0.1414)		
	Hope	0.6471^***^			-0.0426									-0.0280 (-0.1352, 0.0923)		
	Optimism	0.6592^***^			-0.3032^***^									-0.1998^*^ (-0.3307, -0.0980)		

* Notes: c: associations of life satisfaction with depressive and anxiety symptoms; a: associations of life satisfaction with PsyCap and its components; b: associations of PsyCap and its components with depressive and anxiety symptoms after controlling for the predictor variables; c’: associations of life satisfaction with depressive and anxiety symptoms after adding PsyCap as mediator; a × b: the product of a and b; BCa 95% CI: the bias-corrected and accelerated 95% confidence interval

Sex was covariate

*p < 0.05; ** *p* < 0.01; *** *p* < 0.001

## Discussion

The main purpose of this study was to determine the effect of life satisfaction on anxiety and depressive symptoms in Chinese medical students during the COVID-19 pandemic. To the best of our knowledge, this is the first study to show that PsyCap and its components mediate the relationship between life satisfaction and symptoms of depression and anxiety. Research has shown that anxiety and depressive symptoms persist among medical students, who are known to often face high levels of academic stress, especially in the context of the pandemic [36]. Meanwhile, medical students face increasing employment pressures [37]. This may be because medical students believe that if people find them mentally unhealthy, they are perceived as unsuitable therapists by patients with medical problems. In support of this notion, a study found that medical students resolved that they would not disclose their mental health issues for fear of jeopardising their careers [38]. In addition, after medical students enter their third year of university, the increase in clinical professional courses multiplies the pressure, which leads to anxiety and depressive symptoms. All of these factors cause symptoms of anxiety and depression in medical students. Medical students showed more symptoms of depression and anxiety than general college students. Furthermore, there is evidence that the highest amount of stress occurs in the first year and during the transition from the preclinical to clinical stage (i.e. third year) [39]. Therefore, we chose third-year medical students as study participants.

In the current study, we reported the prevalence of anxiety symptom among medical students was 13.2%, and the depressive symptom was 72%, which was higher than that reported in previous studies [40–43]. One reason for the lower prevalence compared to our results may be that most of their studies were conducted before the COVID-19 pandemic. During the COVID-19 pandemic, many medical students were forced to postpone their study plans, and as a result, depressive and anxiety symptoms may be significantly higher during this period than during other periods. We also found that the prevalence of depressive and anxiety symptoms in some studies [44–48] in the context of the pandemic was lower than our results. The participants in this study were third-year medical students. After medical students enter their third year of university, the increase in clinical professional courses multiplies the pressure, which is more likely to lead to anxiety and depressive symptoms.

We analysed the demographic factors associated with anxiety and depressive symptoms, and found that sex had an impact on these symptoms. Our results indicated that male medical students were more susceptible to depressive and anxiety symptoms than their female counterparts. However, many studies have shown that female students are more likely to experience anxiety and depressive symptoms than male students [49, 50]. Nevertheless, our results are consistent with those reported by Gao et al. [51]. Furthermore, a Nigerian study using the Mini International Neuropsychiatric Interviews found that depressive symptoms was twice as common among male college students compared to female college students [52]. However, the current results are consistent with those of Ahmad et al. [53], who found that female students perform better and have less anxiety than male students. Thus, the results of this study are consistent with previous studies. One possible reason is that male students’ negative attitudes towards emotional openness mean that they may be reluctant to use psychological services during college; various anxiety-related factors may be another reason. Therefore, colleges and universities should pay special attention to the psychological state of male students, and encourage them to express their feelings and seek professional assistance if required.

We analysed the effect of life satisfaction on anxiety and depressive symptoms among medical students. The results of the hierarchical linear regression analysis showed that life satisfaction had a significant effect on depressive and anxiety symptoms. Life satisfaction predicted anxiety and depressive symptoms among medical students and had a negative effect on anxiety and depressive symptoms. This finding is in agreement with previous studies [54, 55]. Studies have shown that life satisfaction declines before the long-term worsening of depressive symptoms, suggesting that life satisfaction may be a sensitive marker or an early indicator of the subsequent worsening of psychopathological symptoms [56].

The mechanism underlying the relationship between life satisfaction and depressive and anxiety symptoms requires further study to explore the role of positive predictors and mediators in interventions for depressive and anxiety symptoms. Developing practical strategies for medical students’ PsyCap can help them cope better with a variety of stressors. Life satisfaction was positively correlated with PsyCap, whereas PsyCap and its components (resilience and optimism) were negatively correlated with depressive and anxiety symptoms. This finding is consistent with previous studies [57, 58]. Studies have shown that teachers who are satisfied with their lives are intrinsically optimistic. Active teachers working in private and public schools were more satisfied with their living conditions [59]. Hassan et al. [60] concluded that people satisfied with their lives were more hopeful and positive. Bailey et al. [61] concluded that optimism and hope were correlated with life satisfaction. Appropriate levels of PsyCap can reduce symptoms of anxiety and depression. It has been suggested that people with a higher PsyCap may have a highly adaptive coping mechanism that allows them to successfully combat anxiety and depressive symptoms [62]. Among medical students, optimism and resilience were inversely correlated with anxiety and depressive symptoms, respectively. Resilient individuals show more stable emotions in the face of adversity [63]. Optimism is associated with the attribution of success or a positive outlook, including positive emotions and motivation [63]. PsyCap may mediate the links between life satisfaction, anxiety, and depressive symptoms.

However, our study found that self-efficacy was positively related to anxiety and depressive symptoms, which is inconsistent with the findings of most prior studies [64–66]. Self-efficacy is a person’s positive belief in their ability to successfully complete challenging tasks. People with high self-efficacy can cope effectively with varieties challenges; therefore, they experience lower levels of anxiety and depressive symptoms. From an internal perspective, people with high self-efficacy may have higher expectations of themselves or be more actively engaged in doing things. However, during the pandemic, the pressures regarding studies and life may not be resolved and may be beyond their control, which can increase their level of depressive and anxiety symptoms. Externally, people with high self-efficacy try to solve their problems, but during the pandemic they have less external support from teachers, family, and friends. In particular, our object of study was third-year clinical medical students. Unlike students from other majors, these students must enter the production internship period at this stage. Students with high self-efficacy may want to achieve better results, but due to the pandemic, many learning problems have not been solved, and they cannot achieve their desired goals. Therefore, they are prone to depressive and anxiety symptoms. To the best of our knowledge, this is the first study to examine the mediating role of self-efficacy on Chinese medical students’ life satisfaction, anxiety, and depressive symptoms during a pandemic. Next, we increased the sample size to further verify our results. Therefore, the related inferences must be drawn prudently.

This study is the first to confirm the mediating role of PsyCap and its components in the relationships between life satisfaction, anxiety, and depressive symptoms among Chinese medical students. High life satisfaction may improve PsyCap levels, thereby alleviating depressive and anxiety symptoms in medical students. Among the PsyCap components, self-efficacy, optimism, and resilience partially mediated the relationship between life satisfaction and depressive symptoms in medical students, but completely mediated the relationship between life satisfaction and anxiety symptoms. As mentioned previously, when combining the four PsyCap constructs into a single higher-order construct, PsyCap can be developed in several ways. Thus, our results have a practical significance for Chinese medical students. Based on the results of this study, Chinese medical students should apply previously developed strategies for improving PsyCap to ameliorate their PsyCap levels and alleviate symptoms of depression and anxiety.

Effective strategies should be adopted to improve the life satisfaction and PsyCap of Chinese medical students and alleviate their depressive and anxiety symptoms. Particularly, self-efficacy, optimism, and resilience should be valued in PsyCap investment [67]. Administrators should improve medical students’ life satisfaction and care for their health by establishing fair and equitable procedures, providing adequate praise and rewards to students who perform well, maintaining meaningful communication with medical students, assisting them with learning planning, and offering good learning conditions. To improve medical students’ optimism, administrators should encourage them to use past failures and setbacks as valuable experiences, develop positive attributional styles, and strengthen their ability of medical students to identify and seek various opportunities [63]. To improve resilience, administrators should encourage medical students to utilize their current individual resources, devise solutions to avert or overcome barriers to agreement, and promote critical reflection [68].

### Limitations

Although the findings of this study are valuable, their limitations cannot be ignored. First, because of the lack of a longitudinal study design, we were unable to track the variables related to the students and their medical education processes. Future research should use longitudinal designs to establish the causal relationships between variables. Second, the participants belonged to three medical schools in Liaoning Province, China, which may have limited the generalisability of the findings. Third, in future studies, it is best to conduct detailed interviews rather than using self-reported questionnaires to assess depressive and anxiety symptoms. As both topics are fairly sensitive, many people are reluctant to reveal their thoughts about them, either for fear of stigmatisation or because of an unwillingness to be perceived negatively. Fourth, there is a possibility of a recall bias among the participants. Fourth, increasing the sample size will help increase the generalisability of the results. Finally, our study found that self-efficacy was positively related to anxiety and depressive symptoms, which is inconsistent with the findings of most prior studies. As a part of PsyCap, this inconsistent outcome of self-efficacy may limit the ability to generalize our findings to the post-pandemic era.

## Conclusions

During the COVID-19 pandemic, life satisfaction and PsyCap can act as positive resources for third-year medical students to overcome symptoms of anxiety and depression. Life satisfaction is also a positive resource for developing PsyCap and its four components. Additionally, PsyCap and its components (optimism, resilience, and self-efficacy) partially mediated the association between life satisfaction and depressive symptoms, and fully mediated the link between life satisfaction and anxiety symptoms. Therefore, life satisfaction and PsyCap (especially self-efficacy, resilience, and optimism) should be incorporated into the prevention and treatment of anxiety and depressive symptoms among third-year Chinese medical students during the COVID-19 pandemic or other similar disadvantages contexts. At the same time, additional attention is needed to pay for self-efficacy in such disadvantageous contexts. Our findings can contribute to the development of specialised mental health programmes to screen for, prevent, and treat depressive and anxiety symptoms among college students. Colleges and universities need to assign more resources to the surveillance and early discovery of distress among medical students. Medical education providers should also be encouraged to provide appropriate psychological support for students.

## Electronic supplementary material

Below is the link to the electronic supplementary material.

Supplementary Material 1: Table A. Mediating roles of PsyCap and its components on the Life satisfaction-Depressive/Anxiety symptoms association

## Data Availability

The datasets used and/or analysed during the current study available from the corresponding author on reasonable request.
