# Supplementary material for: The mediating role of psychological capital in the association between life satisfaction and depressive and anxiety symptoms among Chinese medical students during the COVID-19 pandemic: a cross-sectional study
Source: BMC Psychiatry. 2023 Jun 5;23:398. doi: 10.1186/s12888-023-04894-7 (PMC10240134; doi:10.1186/s12888-023-04894-7)
Supplement: Supplementary file 1 — Supplementary Material 1: Table A. Mediating roles of PsyCap and its components on the Life satisfaction-Depressive/Anxiety symptoms association [file 12888_2023_4894_MOESM1_ESM.docx]

**Table A.** Mediating roles of PsyCap and its components on the Life satisfaction-Depressive/Anxiety symptoms association

|  | Mediators | a |  | | | b |  | | c | c’ | | | | a×b (BCa 95% CI) | | | R^2^ |
| --- | --- | --- | --- | --- | --- | --- | --- | --- | --- | --- | --- | --- | --- | --- | --- | --- | --- |
|  |  |  |  | | |  |  | |  |  |  |  |  |  |  |  |  |
| Depression |  |  |  | | |  |  | |  |  | |  | | | |  |  |
| (Score ≥ 16) | PsyCap | 0.5305^***^ | |  |  | -0.2173^***^ | | -0.1526^***^ | | -0.0374 |  | |  | | -0.1124^*^ (-0.1846, -0.0616) |  | 0.0935 |
|  | Self-efficacy | 0.4732^***^ | |  |  | 0.1494^**^ | | -0.1526^***^ | | -0.0269 |  | |  | | 0.0747^*^ (0.0082, 0.1412) |  | 0.2188 |
|  | Resilience | 0.3023^***^ | |  |  | -0.3336^***^ | |  |  |  |  | |  | | -0.1003^*^ (-0.1558, -0.0602) |  |  |
|  | Hope | 0.5168^***^ | |  |  | 0.0241 | |  |  |  |  | |  | | 0.0098 (-0.0719, 0.1284) |  |  |
|  | Optimism | 0.5194^***^ | |  |  | -0.2080^**^ | |  |  |  |  | |  | | -0.1055^*^ (-0.2424, -0.171) |  |  |
| Anxiety  (Score ≥ 50) |  |  | |  |  |  | |  | |  |  | |  | |  |  |  |
|  | PsyCap | 0.4871^***^ | |  |  | -0.0119 | | 0.1756^**^ | | 0.1814^**^ |  | |  | | -0.2749^*^ (-0.1090, 0.0965) |  | 0.1274 |
|  | Self-efficacy | 0.4351^***^ | |  |  | 0.1921^**^ | | 0.1756^**^ | | 0.2628^***^ |  | |  | | 0.1036 (-0.0081, 0.2687) |  | 0.4186 |
|  | Resilience | 0.2285^**^ | |  |  | -0.1013 | |  |  |  |  | |  | | -0.0326 (-0.1155, 0.0198) |  |  |
|  | Hope | 0.4808^***^ | |  |  | 0.1764 | |  |  |  |  | |  | | 0.0561 (-0.0128, 0.3684) |  |  |
|  | Optimism | 0.5175^***^ | |  |  | -0.4491^***^ | |  |  |  |  | |  | | -0.2156^*^ (-0.5103, -0.0275) |  |  |

Notes: c: associations of life satisfaction with depressive and anxiety symptoms; a: associations of life satisfaction with PsyCap and its components; b: associations of PsyCap and its components with depressive and anxiety symptoms after controlling for the predictor variables; c’: associations of life satisfaction with depressive and anxiety symptoms after adding PsyCap as mediator; a × b: the product of a and b; BCa 95% CI: the bias-corrected and accelerated 95% confidence interval.

Sex was covariate.

**p* < 0.05; ** *p* < 0.01; *** *p* < 0.001.
